# Menopause induces changes to the *stratum corneum* ceramide profile, which are prevented by hormone replacement therapy

**DOI:** 10.1038/s41598-022-26095-0

**Published:** 2022-12-15

**Authors:** Alexandra C. Kendall, Suzanne M. Pilkington, Jonathan R. Wray, Victoria L. Newton, Christopher E. M. Griffiths, Mike Bell, Rachel E. B. Watson, Anna Nicolaou

**Affiliations:** 1 Laboratory for Lipidomics and Lipid Biology, Division of Pharmacy and Optometry, School of Health Sciences, The University of Manchester, 2.019C Stopford Building, Oxford Rd, Manchester, M13 9PT UK; 2 Division of Musculoskeletal and Dermatological Sciences, Centre for Dermatology Research, School of Biological Sciences, Faculty of Biology, Medicine, and Health, The University of Manchester, Manchester, UK; 3 The No7 Beauty Company, Boots UK Ltd, Nottingham, UK; 4 National Institute of Health Research Manchester Biomedical Research Centre, Manchester University NHS Foundation Trust, Manchester Academic Health Science Centre, Manchester, UK; 5 Lydia Becker Institute of Immunology and Inflammation, Faculty of Biology, Medicine and Health, The University of Manchester, Manchester, UK

**Keywords:** Lipidomics, Lipids, Menopause

## Abstract

The menopause can lead to epidermal changes that are alleviated by hormone replacement therapy (HRT). We hypothesise that these changes could relate to altered ceramide production, and that oestrogen may have a role in keratinocyte ceramide metabolism. White Caucasian women were recruited into three groups: pre-menopausal (n = 7), post-menopausal (n = 11) and post-menopausal taking HRT (n = 10). Blood samples were assessed for hormone levels, transepidermal water loss was measured to assess skin barrier function, and *stratum corneum* lipids were sampled from photoprotected buttock skin. Ceramides and sphingomyelins were analysed by ultraperformance liquid chromatography with electrospray ionisation and tandem mass spectrometry. Post-menopausal *stratum corneum* contained lower levels of ceramides, with shorter average length; changes that were not evident in the HRT group. Serum oestradiol correlated with ceramide abundance and length. Ceramides had shorter sphingoid bases, indicating altered de novo ceramide biosynthesis. Additionally, post-menopausal women had higher sphingomyelin levels, suggesting a possible effect on the hydrolysis pathway. Treatment of primary human keratinocytes with oestradiol (10 nM) increased production of CER[NS] and CER[NDS] ceramides, confirming an effect of oestrogen on cutaneous ceramide metabolism. Taken together, these data show perturbed *stratum corneum* lipids post-menopause, and a role for oestrogen in ceramide production.

## Introduction

The menopause occurs when a decline in ovarian follicular activity leads to the cessation of menstruation and a loss of fertility^1^. The associated reduction in levels of reproductive hormones, particularly oestrogen, causes additional non-reproductive symptoms that include changes to the metabolism, central nervous system, and skin^2^. Cutaneous changes include skin thinning^3^, reduced elasticity^4^, and impaired wound healing^5^, all of which can be linked to dermal changes such as age-related reductions in collagen production^6–8^. However, other skin changes, such as skin sensitivity^9^, reduced *stratum corneum* (SC) cohesion^10^, and changes to epidermal structure and biomechanical function^11^ point to a dysfunctional epidermal barrier.

The SC forms part of the epidermal barrier, and comprises terminally-differentiated keratinocytes (corneocytes) embedded in a lipid matrix consisting primarily of ceramides, cholesterol and free fatty acids^12^. The ceramides are a complex class of bioactive lipids, and the abundance and quality of ceramides produced by keratinocytes affects the structure and function of the epidermal barrier. Indeed, ceramide abnormalities are found in many skin conditions that feature a dysfunctional epidermal barrier, including atopic dermatitis, psoriasis and acne^13–19^. Therefore, changes to ceramide composition, via altered production and/or metabolism may underlie some of the epidermal changes observed following menopause. Additionally, multiple studies have reported that hormone replacement therapy (HRT), either topical or systemic, can improve menopause-related skin problems^3,4,9,11,20–23^, indicating that the changes are likely directly related to hormonal changes, rather than a consequence of chronological ageing. There have been some limited studies on circulating ceramides in post-menopausal women, but the effect of HRT was not examined^24,25^, and menopause-induced changes to the epidermal ceramide profile remain unexplored.

**Figure 1:**
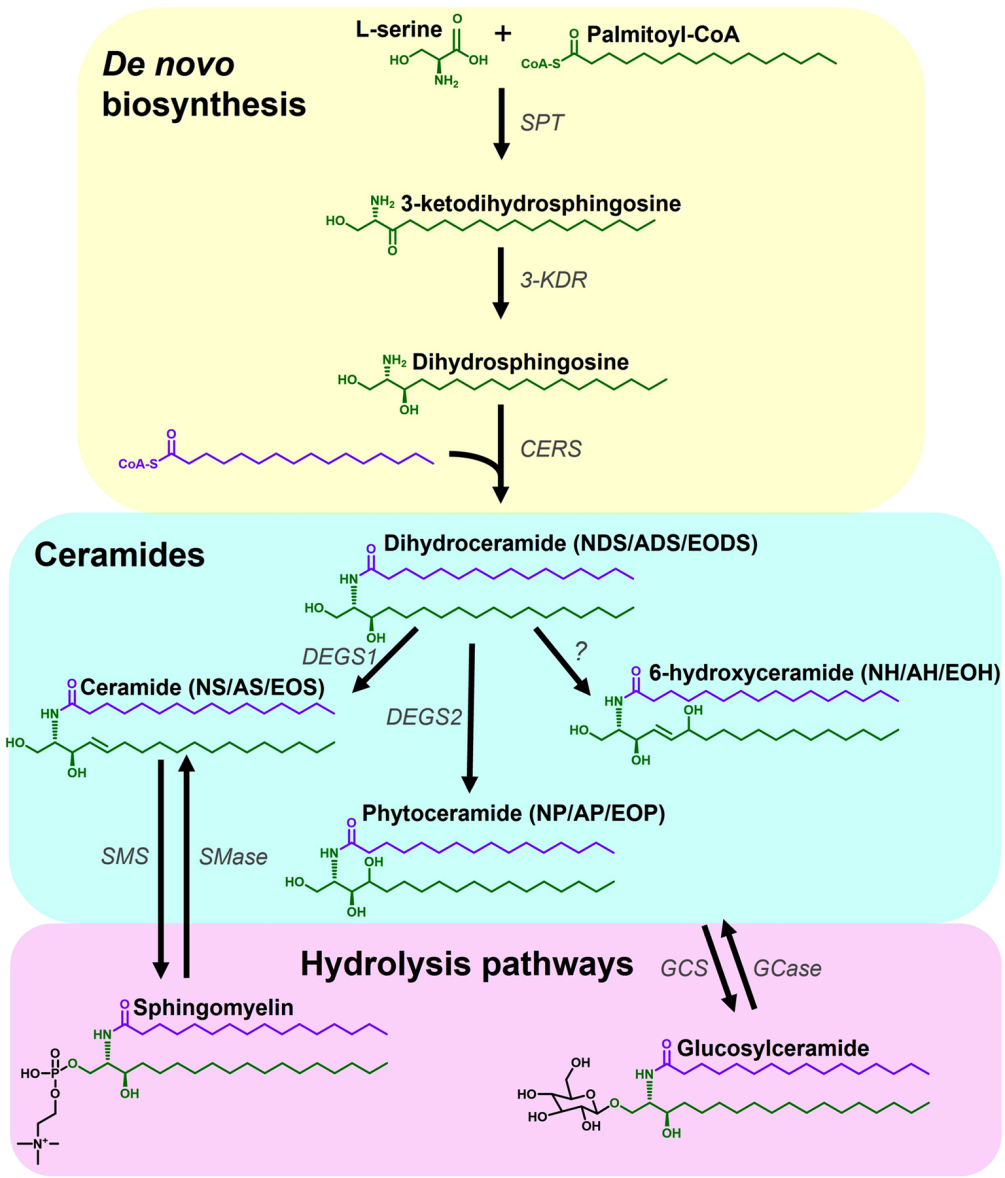
Summary diagram showing parts of the ceramide synthesis pathway. Ceramides are synthesised de novo via the combination of a sphingoid base, generated by the condensation of l-serine and an acyl-CoA, with an acyl chain, via the action of ceramide synthase enzymes. The dihydroceramide formed can be converted to different ceramide classes via the action of dihydroceramide desaturases. Ceramides can be reversibly converted to sphingomyelins (CER[NS/AS]) or glucosylceramides via the main hydrolysis pathways. Alterations in the de novo and hydrolysis pathways can alter the levels of ceramides measured. *3-KDR* 3-ketodihydrosphingosine reductase, *AH* alpha-hydroxyceramide with 6-hydroxysphingone base, *AP* alpha-hydroxyceramide with phytosphingosine base, *AS* alpha-hydroxyceramide with sphingosine base, *CERS* ceramide synthases, *DEGS1* dihydroceramide desaturase 1, *DEGS2* dihydroceramide desaturase 2, *EODS* ester-linked omega hydroxy dihydroceramide, *EOH* ester-linked omega hydroxy 6-hydroxyceramide, *EOP* ester-linked omega hydroxy phytoceramide, *EOS* ester-linked omega hydroxyceramide, *GCase* glucosylceramidase, *GCS* glucosylceramide synthase, *NH* non-hydroxyceramide with 6-hydroxysphingone base, *NP* non-hydroxyceramide with phytosphingosine base, *NS* non-hydroxyceramide with sphingosine base, *SMase* sphingomyelinase, *SMS* sphingomyelin synthase, *SPT* serine palmitoyltransferase, *?* as-yet unidentified desaturase enzyme.

The ceramides are a diverse class of lipids, each species comprising a sphingoid base amide-linked to an acyl chain (Fig. 1). The epidermis has a unique ceramide profile, and different combinations of sphingoid base (including sphingosine (S), dihydrosphingosine (DS), phytosphingosine (P) and 6-hydroxysphingosine (H)) and acyl chain (including non-hydroxy (N), alpha-hydroxy (A) and ester-linked omega hydroxy (EO)) result in an array of almost 1000 species, with 21 ceramide sub-classes having been identified in human SC to-date^26,27^. The abundance of different ceramide classes influences the formation of the SC lipid matrix, with consequences for epidermal barrier function^28^. Ceramides with an EO acyl chain contain an additional linoleic acid (acylceramides), and are particularly important for epidermal barrier function^26^. Further complexity arises from the diversity of carbon chain lengths of both the sphingoid bases and acyl chains. The chain length of ceramides (also referred to as carbon number), alters the packing and organisation of the lipid multilayer, directly affecting the efficiency of the lipid barrier^29,30^. Therefore, it is important to examine the chain length of the ceramides as well as their abundance in order to appreciate potential changes in the SC barrier functionality.

The primary route of ceramide production is de novo biosynthesis, which begins with the enzyme serine palmitoyltransferase (SPT) that condenses l-serine and an acyl-CoA, typically palmitoyl-CoA, to form the intermediate 3-ketodihydrosphingosine, which is rapidly converted to DS by 3-ketodihydrosphingosine reductase^31^ (Fig. 1). This transferase is a pyridoxal 5′-phosphate (PLP)-dependent α-oxoamine synthase; a heterodimer comprising two subunits, typically SPTLC1 and SPTLC2, requiring both subunits to function, although SPTLC2 is the only one with a PLP-binding motif^31^. However, a third subunit (SPTLC3) has been identified as highly expressed in skin, which can replace SPTLC2 and has a preference for shorter acyl-CoA substrates, generating shorter sphingoid bases^31,32^. Since the SPTLC2 and SPTLC3 confer different acyl-CoA specificities^32^, sphingoid bases of different lengths can be generated, with bases of 14–26 carbons reported in human SC^33^.

Via the action of various ceramide synthases (CerS), DS is acylated to form dihydroceramide (CER[NDS]/CER[ADS]). The CerS (CerS1–6) exhibit preferences for different fatty acyl chain length and degrees of saturation, although CerS3 (the most abundantly expressed in skin along with CerS4, and even more so in differentiated keratinocytes), has a broad range of activity, including ultra-long chain fatty acids^34,35^. The action of these enzymes results in production of ceramides with different acyl chain lengths^35^. The dihydroceramides can be converted to ceramides (CER[NS]/CER[AS]), phytoceramides (CER[NP]/CER[AP]) and 6-hydroxyceramides (CER[NH[/CER[AH]) by the action of dihydroceramide desaturases (DEGS1, DEGS2 and an as-yet unknown desaturase, respectively) on the DS base^36^. Acylceramide synthesis follows the same path, but CerS3 adds an ω-hydroxy ultra-long-chain acyl-CoA to the DS base to form an ω-hydroxydihydroceramide, (the base of which can then be converted by dihydroceramide desaturases to form other ω-hydroxyceramides), followed by the addition of linoleic acid taken from a triacylglycerol by the transacylase enzyme PNPLA1 to produce an acylceramide (CER[EODS]/CER[EOS]/CER[EOP]/CER[EOH])^37^. Ceramides can also be generated by the hydrolysis of complex sphingolipids, including sphingomyelins (SM) and glucosylceramides, which act as ceramide storage molecules^38,39^. Changes to any of these sphingolipid metabolic pathways would lead to an altered ceramide profile^26^, and we hypothesise that ceramide pathway changes could explain some of the epidermal barrier dysfunction observed post-menopause; analysis of cutaneous ceramides in post-menopausal women could reveal which pathway is involved.

To date, there have been no studies examining the relationship between the menopause, HRT and the unique profile of cutaneous ceramides, and so we conducted a targeted lipidomic study exploring the SC ceramide profile in pre-menopausal women, post-menopausal women, and post-menopausal women taking HRT. To examine a potential direct effect of oestrogen on ceramide production we have also conducted a small in vitro study with primary human keratinocytes, measuring changes in the ceramides produced following oestrogen treatment. Our study aims to elucidate the contribution of oestrogen to the production of a healthy SC ceramide profile, and has the potential to show how the loss of hormones due to menopause can lead to changes that may contribute to a defective epidermal barrier.

## Results

### Menopause reduces the abundance and length of stratum corneum ceramides

In a small pilot study we demonstrated that the sample of SC obtained with five consecutive tape strips provided the same information on the ceramide profile as sampling deeper into the SC (Supplementary Fig. S1), in agreement with recent reports^40^. Therefore, for the main study, five tape strips were used to sample SC ceramides, with a third of each tape being pooled to give the optimal amount of sample per volunteer. The amount of protein isolated from the tapes in the main study did not differ significantly between groups ((Pre 51.8 ± 12.6 µg; Post 55.1 ± 14.1 µg; Post + HRT 54.5 ± 16.5 µg).

**Figure 2:**
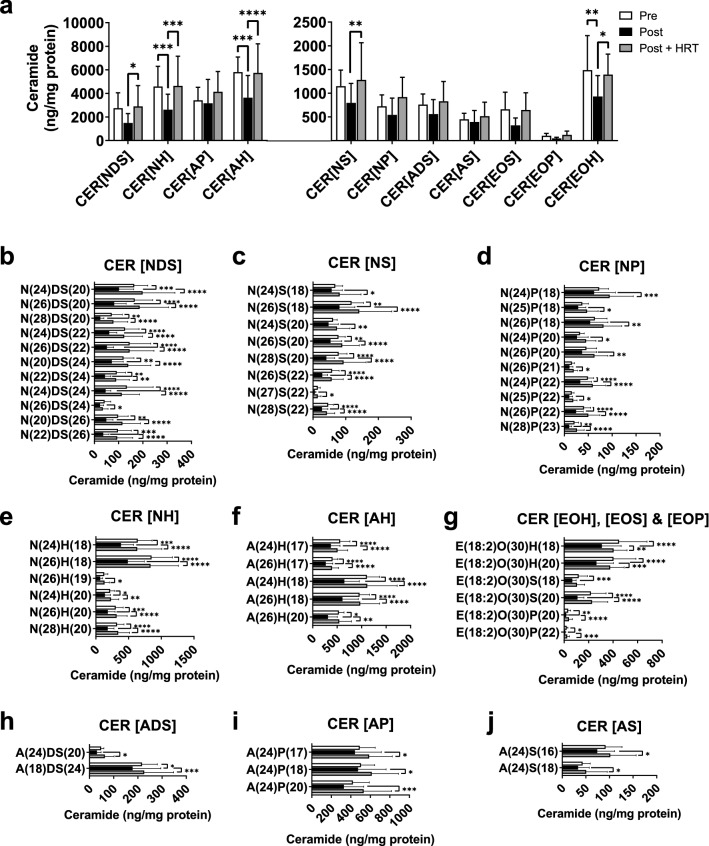
Menopause decreases the abundance of ceramides in the stratum corneum, a change that is prevented by HRT. Individual ceramides were analysed and quantitated by ultraperformance liquid chromatography with electrospray ionisation and tandem mass spectrometry then grouped by class (**a**). Individual species with statistically significant inter-group differences are presented by class (**b**) CER[NDS], (**c**) CER[NS], (**d**) CER[NP], (**e**) CER[NH], (**f**) CER [AH], (**g**) CER[EOH], CER[EOS], and CER[EOP], (**h**) CER[ADS], (**i**) CER[AP], (**j**) CER[AS]. White bars, pre-menopausal (**Pre**, n = 7); black bars, post-menopausal (**Post**, n = 11); grey bars, post-menopausal with hormone replacement therapy (**Post + HRT**, n = 10). Data were normalised against protein content and are presented as mean ± SD. *P < 0.05, **P < 0.01, ***P < 0.001, ****P < 0.0001. One-way ANOVA with Tukey’s multiple comparison, adjusted P values represented.

Post-menopausal women demonstrated lower ceramide abundance compared with the pre-menopausal group, but post-menopausal women taking HRT had levels similar to the pre-menopausal group (Fig. 2). This effect was apparent when levels of individual ceramide species were grouped together to give class totals, with CER[NH], CER[AH] and CER[EOH] achieving statistical significance (P = 0.0008, P = 0.0002 and P = 0.007, respectively; Fig. 2A). Importantly, individual ceramide species from all ceramide classes demonstrated significant reductions in post-menopausal women, which were not evident in the post-menopausal women taking HRT (Fig. 2B–J).

### Menopause reduces the average chain length of stratum corneum ceramides

**Figure 3:**
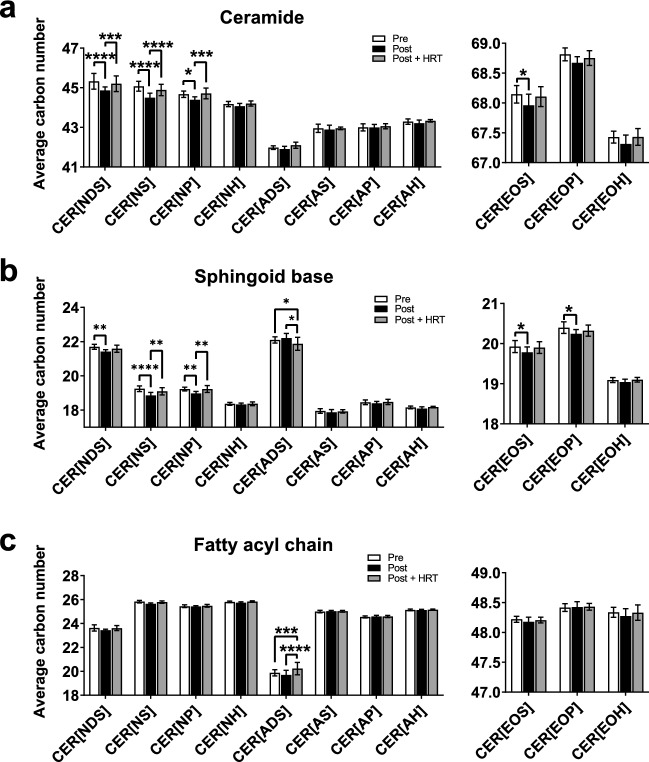
Menopause reduces average ceramide and sphingoid base carbon number in the stratum corneum, changes that are prevented by HRT. Individual ceramides were analysed and quantitated by ultraperformance liquid chromatography with electrospray ionisation and tandem mass spectrometry then grouped by class and average total carbon number was calculated (**a**). Average carbon numbers of sphingoid base (**b**) and acyl chain (**c**) were also calculated per ceramide class. White bars, pre-menopausal (**Pre**, n = 7); black bars, post-menopausal (**Post**, n = 11); grey bars, post-menopausal with hormone replacement therapy (**Post + HRT**, n = 10). Data are presented as mean ± SD. *P < 0.05, **P < 0.01, ***P < 0.001, ****P < 0.0001. One-way ANOVA with Tukey’s multiple comparison, adjusted P values represented.

In addition to their abundance, the quality of ceramides is also important to the SC structure, with shorter ceramides leading to impaired barrier function^16,29,41^. The average total carbon number of ceramides was lower in post-menopausal women, with significant reductions in the total carbon number of the CER[NDS], CER[NS], CER[NP] and CER[EOS] ceramide classes (P < 0.0001, P < 0.0001, P = 0.01 and P = 0.02, respectively; Fig. 3A). Since ceramides comprise a sphingoid base and an acyl chain, both of which vary in length (Fig. 1), the carbon numbers of each component were analysed separately. This analysis revealed that the changes found in SC ceramides post-menopause were due to shorter sphingoid bases, while the length of the acyl chain was largely unaffected by menopause (Fig. 3B,C).

**Figure 4:**
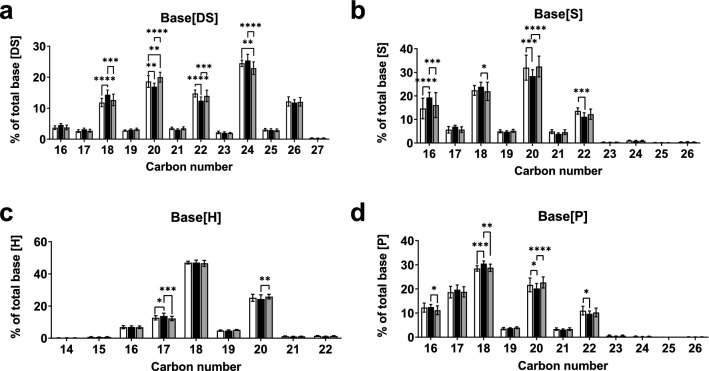
Menopause-induced reduction in sphingoid base carbon number occurs in all types of sphingoid base. Menopause stratum corneum demonstrated increased percentage of shorter sphingoid bases and decreased percentage in longer sphingoid bases across all base types (**a**, dihydrosphingosine (DS); **b**, sphingosine (S); **c**, 6-hydroxysphingosine (H); and **d**, phytosphingosine (P). White bars, pre-menopausal (**Pre**, n = 7); black bars, post-menopausal (**Post**, n = 11); grey bars, post-menopausal with hormone replacement therapy (**Post + HRT**, n = 10). Data are presented as percent of total for respective sphingoid base, mean ± SD. *P < 0.05, **P < 0.01, ***P < 0.001. One-way ANOVA with Tukey’s multiple comparison, adjusted P values represented.

To further investigate what had driven this reduction in average carbon number of the sphingoid bases in post-menopausal women, and examine whether a preference for or scarcity of a particular length of base was responsible for the observed changes, the distribution of different chain lengths within each type of sphingoid base was assessed. There was no single carbon number responsible for the shift in average sphingoid base length and there was an overall shift towards a higher proportion of shorter sphingoid bases of all types in post-menopausal women, compared with pre-menopausal women and post-menopausal women taking HRT (Fig. 4).

### Menopause increases abundance of sphingomyelins but reduces their total carbon number

**Figure 5:**
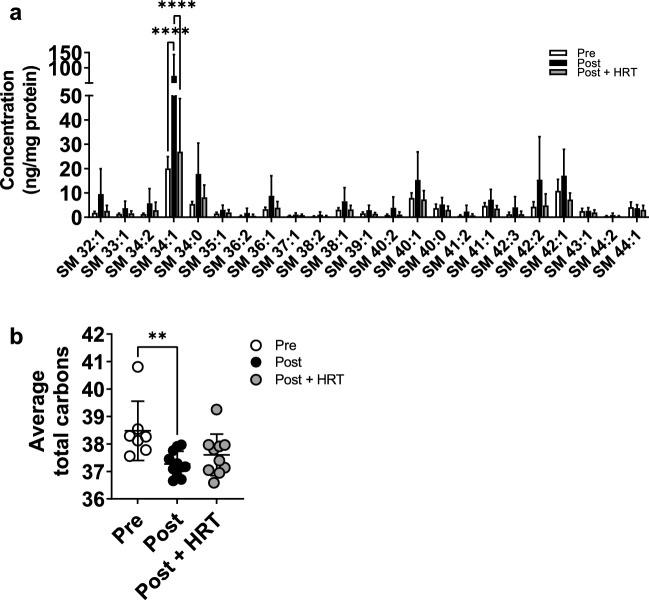
Menopause increases the abundance of sphingomyelins in the stratum corneum, a change that is prevented by HRT, but decreases the average carbon number. Sphingomyelins were analysed and quantitated by ultraperformance liquid chromatography with electrospray ionisation and tandem mass spectrometry (**a**). Average sphingomyelin carbon number was also calculated (**b**). White bars/circles, pre-menopausal (**Pre**, n = 7); black bars/circles, post-menopausal (**Post**, n = 11); grey bars/circles, post-menopausal with hormone replacement therapy (**Post + HRT**, n = 10). Data are presented as mean ± SD. **P < 0.01, ****P < 0.0001. Sphingomyelin concentrations analysed by one-way ANOVAs with Tukey’s multiple comparison, adjusted P values represented, average carbon number analysed by Kruskal–Wallis.

Although changes in sphingoid base length indicate disruption of the de novo biosynthesis pathway, the reduced abundance of SC ceramides following menopause could also arise from their increased storage in complex sphingolipids, including SM and glucosylceramides. Analysis of SM species revealed that post-menopausal women had an overall higher abundance than pre-menopausal women, and that the HRT group was not affected in the same way (Fig. 5A). This was significant for the most abundant SM species with 34 carbons and one double bond (SM 34:1; P < 0.0001), but the trend was clear across the range of SM species analysed. This shows that post-menopause more ceramides are stored as SM, which could partially explain the reduced abundance of SC ceramides. In addition, the average total carbon number of these SM species was lower in post-menopausal women not taking HRT (P = 0.0082; Fig. 5B), which could reflect the uptake of shorter ceramides produced by these women.

### Ceramide length but not abundance correlates negatively with transepidermal water loss

**Figure 6:**
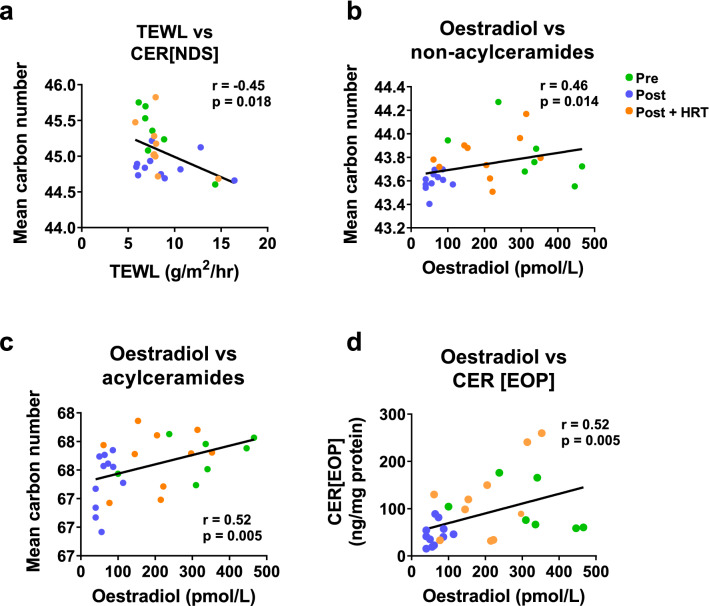
Ceramide length correlates negatively with TEWL and positively with serum oestradiol, whilst serum oestradiol correlates positively with amount of ceramides. Pearson correlation coefficients were calculated using data from all three groups of volunteers between TEWL and CER[NDS] carbon number (**a**), serum oestradiol and non-acylceramide carbon number (**b**), serum oestradiol and acylceramide carbon number (**c**) and serum oestradiol and CER[EOP] (**d**). Green circles, pre-menopausal (**Pre**, n = 7); purple circles, post-menopausal (**Post**, n = 11); orange circles, post-menopausal with hormone replacement therapy (**Post + HRT**, n = 10).

To explore the impact of SC ceramides on barrier function, TEWL was assessed, and found to correlate negatively with the average total carbon number of CER[NDS] (Fig. 6A), reflecting the fact that shorter ceramides form a less effective epidermal barrier, allowing more water loss in post-menopause skin.

### Ceramide abundance and length correlate with serum oestradiol

Since menopause-induced changes in ceramides were absent or reduced in the HRT group, we assessed the relationship between serum female hormone levels and SC ceramides. Ceramide total carbon number, in both acylceramides (CER[EOS]/CER[EOH/CER[EOP]) and non-acylceramides (all other classes measured), correlated positively with serum oestradiol (Fig. 6B,C), demonstrating that volunteers with higher circulating oestradiol levels also had longer SC ceramides, with post-menopausal volunteers having lower oestradiol levels than the other groups. Serum oestradiol also correlated positively with the abundance of SC CER[EOP] (Fig. 6D), showing that the women with higher circulating oestradiol levels also had higher levels of SC ceramides.

### Oestradiol increases keratinocyte ceramide production in vitro

**Figure 7:**
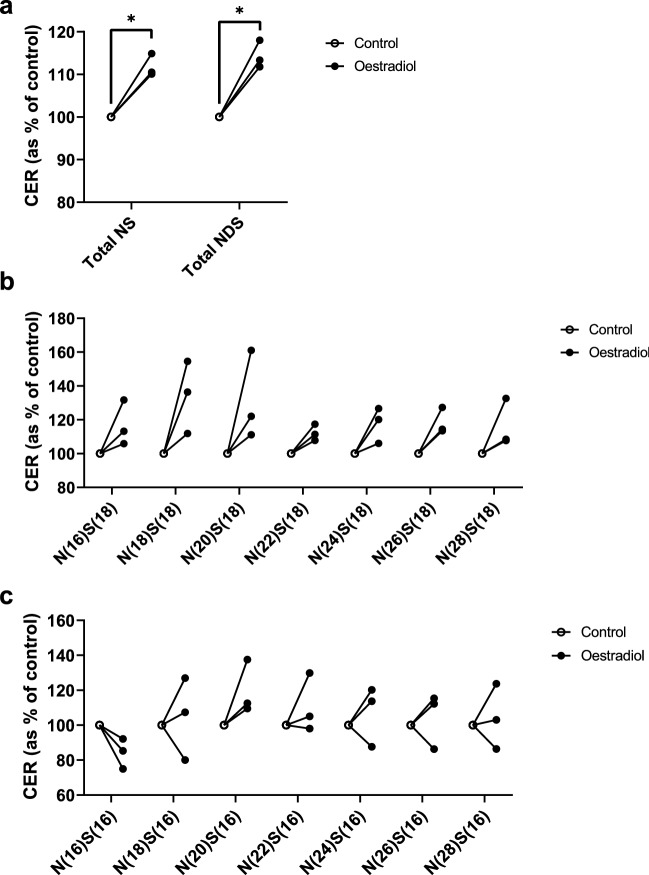
Oestradiol provided exogenously to differentiating primary human keratinocytes increases production of CER[NS] and CER[NDS]. Differentiating keratinocytes (cultured in 1.8 mM Ca^2+^) were treated with oestradiol (10 nM, 72 h). Individual ceramides were then extracted and analysed by ultraperformance liquid chromatography with electrospray ionisation and tandem mass spectrometry and grouped by ceramide class. Total CER[NS] and CER[NDS] are presented (**a**) as well as individual ceramides with a 18-carbon sphingosine base (**b**) and a 16-carbon sphingosine base (**c**). Data are presented as percentage of untreated control for each keratinocyte donor. *P < 0.05, analysed by paired *t* tests corrected for multiple testing using the Holm–Šídák method, n = 3 different keratinocyte donors.

The correlation between serum oestradiol and SC ceramides supports the idea that exogenous provision of hormones could be responsible for the restoration of a normal SC ceramide profile observed in post-menopausal women taking HRT. To investigate the causal relationship between reproductive hormones and epidermal ceramide production, primary normal human epidermal keratinocytes (NHEK) isolated from the skin of female donors were differentiated in vitro to synthesise epidermal ceramides. The most abundant ceramide classes produced by keratinocytes in vitro (CER[NS] and CER[NDS]) demonstrated increased production following treatment with exogenous oestradiol, indicating a direct relationship between oestradiol and ceramide metabolism (Fig. 7A and Supplementary Fig. S2). Since changes in sphingoid base carbon numbers were observed post-menopause but not in the HRT group, NS ceramides with C16 and C18 bases were compared (Fig. 7B,C). CER[NS] with a C18 base showed consistent upregulation in response to oestradiol (Fig. 7B), whilst those with the same acyl chain but a C16 base did not rise consistently in all donors, pointing to a potential oestrogen-related shift to longer chains (Fig. 7C). There was no effect of oestradiol on the length of the acyl chain component of the ceramides produced in vitro (data not shown).

## Discussion

Using a targeted lipidomic approach, we found reductions in both the abundance and quality of SC ceramides produced by post-menopausal skin. These changes were absent or reduced in the skin of participants taking HRT, suggesting a direct role for oestrogen in the regulation of the epidermal barrier, and a possible explanation for some of the skin changes reported by women following the menopause^2^.

Aiming to gain mechanistic insight into the observed changes, we explored the composition of ceramides produced (including carbon numbers of sphingoid bases and acyl chains), and these indicated an oestrogen-related change to the de novo biosynthesis pathway. This finding was confirmed by an in vitro pilot study using NHEK, in which oestradiol directly augmented ceramide production. The effect of oestrogen is not simply a result of increased keratinocyte differentiation, (which would increase global ceramide production), since studies have shown that oestrogen actually inhibits keratinocyte differentiation^42,43^, but may point to a specific role for oestrogen in the ceramide metabolic pathway. We have recently reported that analysis of epidermal lipids from biopsies revealed an increase in cholesterol post-menopause, and no change in free fatty acid levels, showing that the menopause-related reduction in ceramides is not simply due to a global decrease in lipid production^11^. These observations indicate that oestrogen induces a specific disturbance in the ceramide metabolic pathway at a point that affects all types of ceramides, potentially via the de novo pathway or increased uptake/storage in complex molecules such as SM and glucosylceramides (Fig. 1).

We observed a decrease in the abundance of SC ceramides in post-menopausal women, which was not apparent in post-menopausal women taking HRT. Slight differences between buttock ceramide concentrations reported in our study and others^44^ could result from technical differences, such as the type of internal standards used in each study, and different numbers of ceramides examined in each class targeted for analysis, overall leading to differences in class totals. Furthermore, SC ceramide profiles vary across the body, as reflected in the differences observed between the buttock and cheek samples, analysed in our study (Fig. 2 and Fig. S1)^44–46^. Additionally, the ceramide profile in the SC of our unique cohort of females, around the age of menopause, could be influenced by factors such as changes in reproductive hormones (not limited to the oestradiol measured in our study), and an age-related increase in skin pH and decrease in sebum production^47^. Sebum production has been shown to influence the SC ceramide profile at different anatomical sites^48^, and this likely extends to chronological changes in sebum production. Despite differences between studies, the relative changes within our study show a clear effect of menopause on ceramides.

The ceramide pathway is a complex, tightly-controlled metabolic system (Fig. 1) comprising de novo biosynthesis as well as storage and recycling of ceramides via complex sphingolipids including SM and glucosylceramides; levels of ceramides can be influenced by alterations to several aspects of this pathway. Since we observed changes to all ceramide classes, it is likely that a single enzyme upstream of dihydroceramide production is affected by menopause, most likely SPT, which synthesises the sphingoid base, or a range of CerS, which provide the acyl chain^26^. Since we observed no change in the average length of the acyl chains, which would indicate a change in activity of specific CerS, it is unlikely that insufficient acyl chain provision led to the decrease in ceramide abundance. Additionally, our previous analysis of epidermal lipids, which reflects the lipids available for ceramide synthesis, showed no change in free fatty acid levels post-menopause^11^. Indeed, the rate-limiting step of ceramide synthesis is the activity of SPT^26^. The regulation of SPT expression and activity has not been fully elucidated, although proteins called *small subunits of SPT* and *orosomucoid-like proteins* have been identified that act as regulatory components of the SPT complex, enhancing and reducing its activity, respectively^49,50^. It is possible that oestrogen influences the expression or activity of these regulatory proteins, thereby altering the activity of SPT, although little is known about what controls these regulatory proteins^49^.

Although the de novo pathway is a likely route of the change in ceramide abundance, the observed increase in SM abundance in post-menopausal women could also result from a shift towards storage of ceramides (Fig. 1). Although both the SM and glucosylceramide hydrolysis pathways could influence ceramide abundance, our targeted ceramide analysis requiring specific extraction and mass spectrometry assays left only enough sample to explore one. Evidence from a reconstructed skin model suggests oestrogen could influence the SM pathway^51^. Furthermore, there is an increase in skin pH at the time of the menopause that could impair the activity of the SM hydrolytic enzyme acid sphingomyelinase^47,52^, while research shows that the SM-to-ceramide ratio has consequences for the permeability of the SC barrier^53^. Therefore, we chose to analyse SM levels in our samples. However, SM are known to store and release only CER[NS] and CER[AS], therefore this would not explain the decrease observed in the other ceramide classes^38,52^. Other ceramide classes can be stored as glucosylceramides, which store not just CER[NS] and CER[AS] but all other classes, including CER[EOS] that are crucial for formation of the cornified lipid envelope^39,52,54^. Analysis of glucosylceramides in women pre- and post-menopause could reveal whether this branch of the hydrolysis pathway is also involved, and would be of interest in future studies.

As well as a decrease in the abundance of SC ceramides, menopause caused a change in the total carbon number of the ceramides, reducing their average chain length. Shorter ceramides have previously been identified in conditions featuring epidermal barrier insufficiencies, including atopic dermatitis^15,16^. In these studies the shorter ceramides were associated with increased TEWL and reduced skin capacitance, which are signs of impaired barrier function^15,16^; studies of model membranes have shown that shorter ceramides modify the organisation of lipids in the barrier, increasing permeability of the model membranes^41^. Indeed, the reduction in average total carbon number observed in our study was similar to that seen in the non-lesional skin of atopic dermatitis patients, who also demonstrated impaired SC lipid organisation^16^. For post-menopausal women this could mean their skin is more susceptible to irritants, allergens, and microorganisms, and increased water loss may lead to dry skin. Analysis of the carbon numbers of the ceramide components revealed that the shortening of ceramides in post-menopausal women was caused by a decrease in the average length of the sphingoid base, with no change in acyl chains, again indicating a role for SPT rather than CerS. Given the different acyl-CoA specificities of the SPT subunits SPTLC2 and SPTLC3, it is possible that the loss of oestrogen following menopause alters the composition of SPT subunits, with a preference for SPTLC3 over SPTLC2, resulting in the synthesis of shorter sphingoid bases^31,32^. When oestradiol was added to NHEK, upregulation of CER[NS] with a C18 base was seen, which could result from possible increased expression or activity of SPT with an SPTLC2 subunit (which prefers palmitoyl-CoA as a substrate, making a C18 base), but CER[NS] with a C16 base were not consistently upregulated, suggesting that SPT with an SPTLC3 subunit (which prefers myristoyl-CoA as a subunit, making a C16 base) may not have been promoted to the same degree. Indeed, oestrogen depletion in ovariectomised rats led to decreased aortic expression of SPTLC2 (which was reversed by addition of oestrogen), whilst SPTLC3 was unaffected, which would shift the balance towards production of shorter sphingoid bases (although chain length was not measured in the study)^55^. The NHEK used in our study were isolated from three donors of unknown menopausal status, and this may have led to variability in their response to oestrogen, since oestrogen receptors in skin are expressed at lower levels post-menopause^56^, so it would be of interest to explore the ceramide response to oestrogen in a larger number of donors with confirmed menopausal status.

Rather than an age-related change to skin that happens around the same time as menopause, our study provides further evidence that the described menopausal skin changes are a direct result of the decline in circulating reproductive hormones. The correlations between serum oestradiol and ceramide abundance and quality, the restoration of ceramides to a more pre-menopausal profile following treatment by HRT, and the influence of oestradiol on NHEK ceramide production, together demonstrate a direct relationship between oestrogen and ceramide production. This supports the conclusion that the SC ceramide changes observed in our study are directly related to loss of oestrogen during the menopause. Although ceramide profiles differed slightly between photoprotected buttock skin and facial skin in our study and others^44^, circulating hormones would reach the skin at all anatomical sites^57^. Therefore, although the changes in our study were observed in buttock skin, we expect comparable underlying changes in other body sites, such as the face, despite confounding factors such as photoageing. Indeed, facial skin expresses higher levels of oestrogen receptors so may be more susceptible to oestrogen-related changes^58^.

To-date there has been very limited research into the effect of oestrogen on cutaneous ceramides. A recent study by Takeda et al*.* using a reconstructed human epidermal keratinisation model, found that treatment with an oestrogen receptor agonist led to increased levels of CER[EOS], associated with increased expression of SPTLC2, CerS3, glucosylceramide synthase and acid sphingomyelinase, and reduced expression of SM synthase 2^51^. However, in that study, ceramides were analysed by high performance thin layer chromatography, which does not allow detailed analysis of individual ceramides, and the impact on chain length could not be assessed.

There has been more research into non-cutaneous ceramides post-menopause, and the effect of the menopause and oestrogen on ceramide metabolism appears to differ depending on tissue type. Studies have previously reported increases in plasma ceramides following menopause that could potentially be linked to the increased risk of cardiovascular disease and cognitive impairment post-menopause^24,25^. Both studies excluded post-menopausal women taking HRT so the effects of replacing the hormones could not be assessed. The origin of ceramides circulating in the blood is unclear, although studies in mice indicate the liver as a likely source^59,60^.

An in vitro study on human breast cancer cells found that treatment with oestradiol led to decreased ceramide production^24^. Furthermore, two separate in vivo studies on ovariectomised rats (in which oestrogen levels decline), in which the hypothalamus or aorta were analysed, both found increased production of ceramides, which was reversed by oestrogen treatment^55,61^. These studies show that oestrogen has a different effect on epidermal ceramide metabolism than it does on other cell types. Indeed, the pathways affected seem to be different. In the rat aorta, expression of CerS-2, -4 and -6, but not -1, -3 or -5, were decreased by low oestrogen levels^55^, which would lead to changes in the average length of the acyl chain; something not observed in our study.

One possible reason for the differential effect of oestrogen on skin compared with other tissues is the expression of SPTLC3, the subunit of SPT responsible for the generation of shorter sphingoid bases. The skin expresses very high levels of SPTLC3, so any oestrogen-induced effects on SPTLC3 would have a much greater impact on skin than other tissues^62^. The two other tissues where SPTLC3 is most abundantly-expressed are the uterus and placenta^62^, tissues where oestrogen signalling is very important, and so it is possible that oestrogen may regulate this subunit, although this remains unexplored.

In conclusion, we have shown that SC ceramides are less abundant and shorter in length post-menopause, and that the observed changes are less evident in women taking HRT. This effect likely results from oestrogen regulation of the activity and/or composition of the enzyme SPT and further studies examining this link are of interest. As well as changes to the ceramide biosynthesis pathway, there may also be changes in the storage and release of ceramides, and in this study we identified differences in the SM hydrolysis pathway. However, it is important to also explore the glucosylceramide hydrolysis pathway, since this could influence levels of ceramide classes other than CER[NS] and CER[AS] that are stored as SM^39^. Furthermore, in the present study we have focussed on the role of oestrogen without controlling for the types of HRT the volunteers were taking, it is therefore important to explore the impact of different HRT formats and doses.

## Materials and methods

### Study participants

#### Menopause study

Healthy, White Caucasian, female volunteers were recruited to three study groups: (1) pre-menopausal women who were still having regular periods (n = 7; PRE); (2) post-menopausal women who had not had a period within the last 12 months (n = 11; POST), or (3) post-menopausal women who were receiving HRT in a range of formulations and doses (n = 10, POST + HRT)^11^. Volunteers were aged 39–63 years old, with pre-menopausal women being significantly younger than their post-menopausal counterparts (43.7 ± 3.7 vs 55.1 ± 5.4 years; P < 0.0001). Body mass index did not differ between groups. Menopausal status was confirmed by measurement of circulating oestrogen and follicle-stimulating hormone (FSH), and hormone-induced variability in pre-menopausal women was limited by excluding participants who were taking hormonal contraception, and by collecting data during the second half of their menstrual cycle. To ensure accurate baseline measurements, participants were advised not to apply topical skincare products on the day of the clinic visit. The study was conducted in accordance with the Declaration of Helsinki principles (revised Seoul 2008) and ethical approval was granted by the University of Manchester Research Ethics Committee (reference 2017-2954-4020); all volunteers provided written informed consent. The study was conducted at Salford Royal Teaching Hospital (Salford Royal NHS Foundation Trust).

#### Pilot study

Additionally, a small pilot study was performed to confirm the number of tape strips required to analyse SC ceramides. Volunteers (n = 3) were White Caucasian females (aged 29–36 years old). The study was conducted in accordance with the Declaration of Helsinki principles (revised Seoul 2008) and ethical approval was granted by the Reading Independent Ethics Committee (reference 130813-1); all volunteers provided written informed consent. The study was conducted at Boots PLC, Nottingham.

### Tape strip sampling

Tape strips (22 mm diameter discs; D-Squame; Clinical & Derm, Dallas, TX, USA) were used to sample the SC, applied at a consistent pressure of 225 g/cm^2^ using a D-squame pressure instrument (Clinical & Derm)^63^. In a pilot study, 15 sequential layers were harvested from facial cheek skin and analysed to assess the number of tape strips needed to accurately measure SC ceramides; based on the outcomes of this pilot work (Supplementary Fig. S1), in the main study five layers were harvested from photoprotected buttock skin. Tapes were applied sequentially to the same area of skin, then removed and stored separately at − 80 °C until analysis.

### Transepidermal water loss measurement

Prior to transepidermal water loss (TEWL) measurement, volunteers were allowed to acclimatise to room conditions for 20 min. TEWL was then measured adjacent to the tape strip sampling site using a VapoMeter (Delfin Technologies Ltd, Kuopio, Finland). Three separate measurements were taken and averaged to produce the final reading^64^.

### Analysis of serum sex hormones

Blood samples (5 mL) were taken and allowed to clot for 30 min at room temperature, then centrifuged (2500*g*, 15 min). Serum was removed and analysed for circulating concentrations of oestrogen and FSH in the clinical biochemistry department at Salford Royal Teaching Hospital, using standard protocols.

### Keratinocyte culture and treatment

Primary NHEK (Promocell, Heidelberg, Germany) from three different donors (female, aged 45, 58 and 60) were cultured to 80% confluency then differentiated for 24 h using calcium switch (1.8 mM Ca^2+^)^65^. Cells were then treated with 10 nM oestradiol (Thermo Fisher Scientific, Waltham, Massachusetts, USA) (concentration based on^66^) or vehicle control (ethanol) for 72 h (treatment replenished daily). Cells were washed, harvested by trypsinisation, pelleted, then stored at − 80 °C.

### Extraction and preparation of lipids

#### Stratum corneum ceramides

Tape strips were cut into thirds and a third of each tape strip (sequential tapes 1–5 for the main study, or 1–5, 6–10 and 11–15 for the pilot study) were pooled into a single sample per volunteer. Samples were sonicated in ice-cold methanol for 1 h, with occasional vortexing^67^. Extract was removed, an aliquot retained for protein content analysis, and the remainder spiked with internal standards for each ceramide class (4 ng each): CER [N(16)DS(18)]-*d*9, CER [N(16)S(18)]-*d*9, CER [N(16)P(18)]-*d*9, CER [N(16)H(18)]-*d*9, CER [A(16)DS(18)]-*d*9, CER [A(16)S(18)]-*d*9, CER [A(16)P(18)]-*d*9, CER [A(16)H(18)]-*d*9, CER [E(26)O(18:1)S(18)]-*d*9, and CER [E(26)O(18:1)P(18)]-*d*9 (Avanti Polar Lipids, Alabaster, Alabama, USA). Extracts were dried down under nitrogen and reconstituted in hexane:isopropanol (11:1 v/v). Extracts were semi-purified using solid-phase extraction (100 mg aminopropyl cartridges; Sigma, Poole, United Kingdom) to eliminate matrix effects and eluted in hexane:chloroform:methanol (8:1:1, v/v). Finally, samples were dried under nitrogen and reconstituted in methanol with formic acid (0.1%; v/v). Samples were stored at − 20 °C for up to 7 days awaiting analysis.

#### NHEK ceramides

Cell pellets were homogenised in 2:1:0.75 (v/v/v) chloroform:methanol:water, spiked with internal standards for each ceramide class (4 ng each): CER [N(16)DS(18)]-*d*9, CER [N(16)S(18)]-*d*9, CER [N(16)P(18)]-*d*9, CER [N(16)H(18)]-*d*9, CER [A(16)DS(18)]-*d*9, CER [A(16)S(18)]-*d*9, CER [A(16)P(18)]-*d*9, CER [A(16)H(18)]-*d*9, CER [E(26)O(18:1)S(18)]-*d*9, and CER [E(26)O(18:1)P(18)]-*d*9 (Avanti Polar Lipids, Alabaster, Alabama, USA), and incubated on ice for 30 min. Samples were centrifuged (1500*g*, 5 min, 4 °C), and the pellet removed for protein content analysis. Extracts were dried down under nitrogen and reconstituted in chloroform, then semi-purified using solid-phase extraction (100 mg silica cartridges; Phenomenex, Macclesfield, United Kingdom) to eliminate matrix effects, and eluted with sequential additions of 2:1 (v/v) chloroform:methanol then 2:1 (v/v) chloroform:methanol containing 0.1% formic acid. Finally, samples were dried under nitrogen and reconstituted in methanol with formic acid (0.1%; v/v). Samples were stored at − 20 °C for up to 7 days awaiting analysis.

#### Stratum corneum sphingomyelins

Tape strips were cut into thirds and a third of each tape strip (1–5) was pooled into a single sample per volunteer. Samples were sonicated in ice-cold methanol for 1 h, with occasional vortexing. The extract was removed, an aliquot retained for protein content analysis, and the remainder spiked with 4 ng *N*-palmitoyl-d31-d-erythro-sphingosylphosphorylcholine (SM-*d*31; Avanti Polar Lipids, Alabaster, Alabama, USA), before drying down under nitrogen. Samples were reconstituted in chloroform:methanol:water (1:1:0.9; v/v/v) and centrifuged (3000*g*, 5 min, 4 °C) before the lower organic phase was removed and dried under nitrogen. Lipid residue was reconstituted in methanol (1 mL) and alkaline hydrolysis was performed by addition of 1 M sodium hydroxide (10 µL) and incubation at room temperature for 2 h^68^. The extract was neutralised using 1 M glacial acetic acid, dried under nitrogen, and reconstituted in methanol with formic acid (0.1%; v/v). Samples were stored at − 20 °C for up to 7 days awaiting analysis.

### Analysis of ceramides and sphingomyelins by ultraperformance liquid chromatography with electrospray ionisation and tandem mass spectrometry

Extracted lipids were analysed by multiple reaction monitoring (MRM) using ultraperformance liquid chromatography with electrospray ionisation and tandem mass spectrometry (UPLC/ESI–MS/MS), with an Acquity UPLC pump (Waters, Wilmslow, United Kingdom) coupled to an electrospray ionisation triple quadrupole mass spectrometer (Xevo TQ-S; Waters). Autosampler temperature was 8 °C; column temperature was 30 °C; solvent flow rate was 0.3 mL/min and a BEH C8 1.7 µm 2.1 × 100 mm reverse phase column was used (Waters, Wilmslow, United Kingdom). Solvent gradients used for analysis of ceramides or SM using mobile phase A (0.1% formic acid in LC/MS grade water) and mobile phase B (methanol containing 0.1% formic acid) are described in Supplementary Table S1. Electrospray ionisation was performed in positive mode using the following settings: capillary voltage, 3.5 kV; source temperature, 100 °C; cone voltage, 30 V; desolvation gas temperature, 450 °C. A full list of MRMs and collision energies is provided in Supplementary Tables S2 and S3. Ceramide and SM data were analysed using semi-quantitation against class-specific deuterated internal standards, and normalised against protein content.

### Protein content

Protein content was measured as previously described^69–71^. During lipid extractions, protein pellets (from cell extractions), or aliquots of the methanol extract (from tape strip extractions) were retained and stored at − 20 °C until quantitation using a standard Protein Assay Kit (Bio-Rad, Hercules, CA, USA). Proteins were solubilized using 1 M NaOH and analysed within the linear range of the assay to ensure accuracy.

### Statistical analysis

Statistical analyses were performed using Prism version 9.1.2 (GraphPad Software, La Jolla, CA, USA). Normality tests were performed to determine whether data were from a Gaussian distribution and statistical tests were selected accordingly. In detail: volunteer age data were compared using a Mann–Whitney test; ceramide data were compared using one-way ANOVAs followed by Tukey’s test for multiple comparisons; correlations between SC lipids, TEWL and oestradiol were analysed using either Spearman Rank or Pearson’s R correlations; cell data were compared using paired *t* tests corrected for multiple testing using the Holm–Šídák method. All P values stated are adjusted P values, and an adjusted P value of P < 0.05 was considered significant.

## Supplementary Information

Supplementary Information.

## Data Availability

The datasets generated during the current study are available from the corresponding author on reasonable request.

## Abbreviations

A: Alpha-hydroxy
CER: Ceramide
DS: Dihydrosphingosine
EO: Ester-linked omega hydroxy
FSH: Follicle-stimulating hormone
H: 6-Hydroxysphingosine
HRT: Hormone replacement therapy
MRM: Multiple reaction monitoring
N: Non-hydroxy
P: Phytosphingosine
S: Sphingosine
SC: Stratum corneum
SM: Sphingomyelin
SPT: Serine palmitoyltransferase
TEWL: Transepidermal water loss
