# Supplementary material for: Menopause induces changes to the stratum corneum ceramide profile, which are prevented by hormone replacement therapy
Source: Sci Rep. 2022 Dec 15;12:21715. doi: 10.1038/s41598-022-26095-0 (PMC9755298; doi:10.1038/s41598-022-26095-0)
Supplement: Supplementary file 1 — Supplementary Information. [file 41598_2022_26095_MOESM1_ESM.pdf]

Supplementary material

Menopause induces changes to the *stratum corneum* ceramide profile, which are prevented by hormone replacement therapy

Alexandra C. Kendall[1$], Suzanne M. Pilkington[2$], Jonathan R. Wray[1], Victoria L. Newton[3], Christopher E. M. Griffiths[2,4], Mike Bell[3], Rachel E. B. Watson[2,4] and Anna Nicolaou[1,4,5*]

[1] Laboratory for Lipidomics and Lipid Biology, Division of Pharmacy and Optometry, School of Health Sciences, The University of Manchester, Manchester, United Kingdom.

[2] Division of Musculoskeletal and Dermatological Sciences, Centre for Dermatology Research, School of Biological Sciences, Faculty of Biology, Medicine, and Health, The University of Manchester, Manchester, United Kingdom.

[3] The No7 Beauty Company, Boots UK Ltd, Nottingham, United Kingdom.

[4] National Institute of Health Research Manchester Biomedical Research Centre, Manchester University NHS Foundation Trust, Manchester Academic Health Science Centre, Manchester, United Kingdom.

[5] Lydia Becker Institute of Immunology and Inflammation, Faculty of Biology, Medicine and Health, The University of Manchester, Manchester, United Kingdom.

[$] These authors contributed equally to this work.

* Corresponding author: Division of Pharmacy and Optometry, University of Manchester, 2.019C Stopford Building, Oxford Rd, M13 9PT Manchester, United Kingdom. E-mail: anna.nicolaou@manchester.ac.uk.

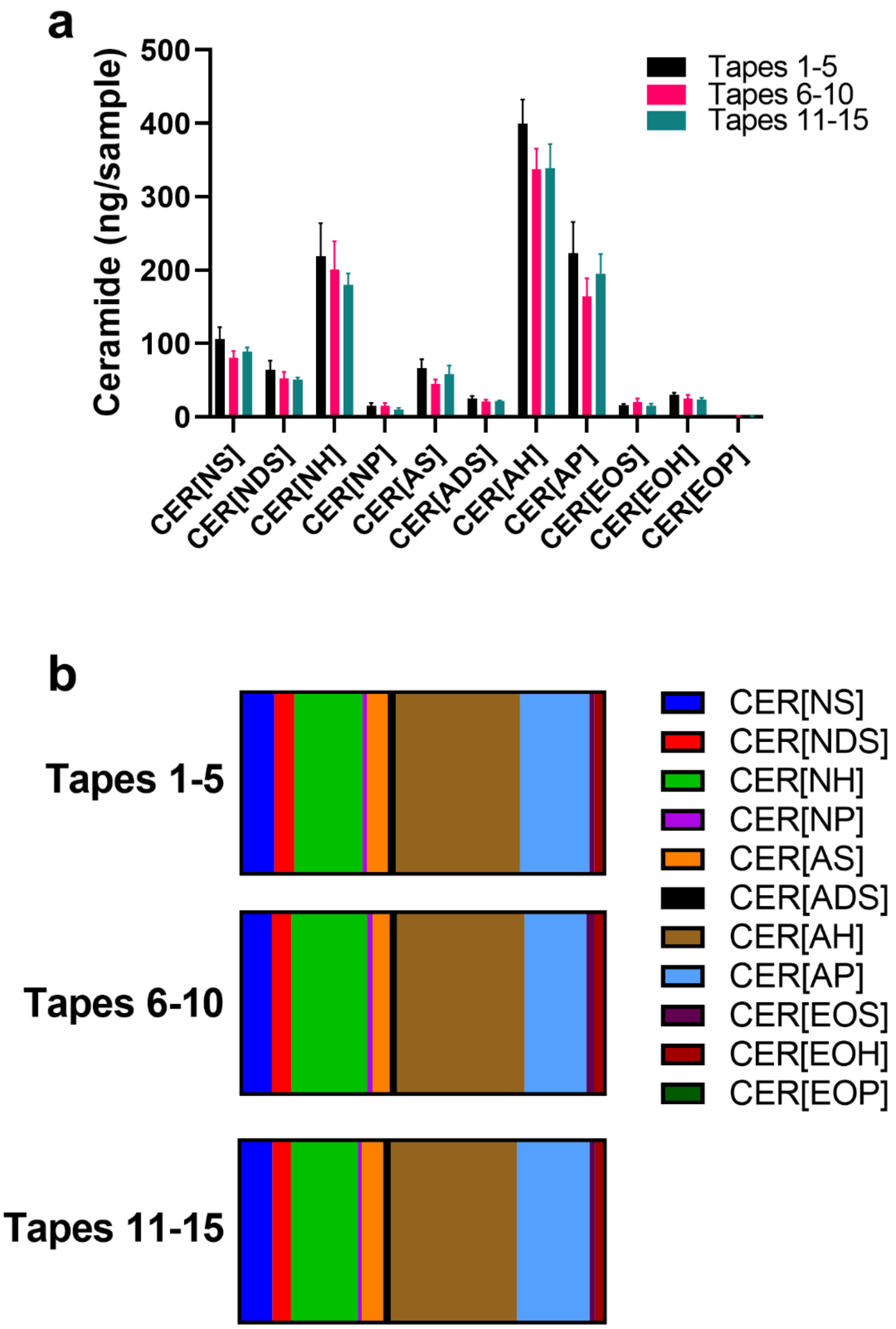

Supplementary Figure S1. Ceramide profile is consistent throughout the first 15 layers of the stratum corneum. Tape strips (15) were taken from facial skin (cheek, in duplicate). A third of each tape was used, with tapes 1-5, 6-10 and 11-15 pooled together for analysis. Ceramides were analysed by ultraperformance liquid chromatography with electrospray ionisation and tandem mass spectrometry, and grouped by ceramide class. Ceramide class data are presented as total amount per pooled sample (**a**), and as percentage of total ceramides (**b**). n=3 donors, two samples per donor.

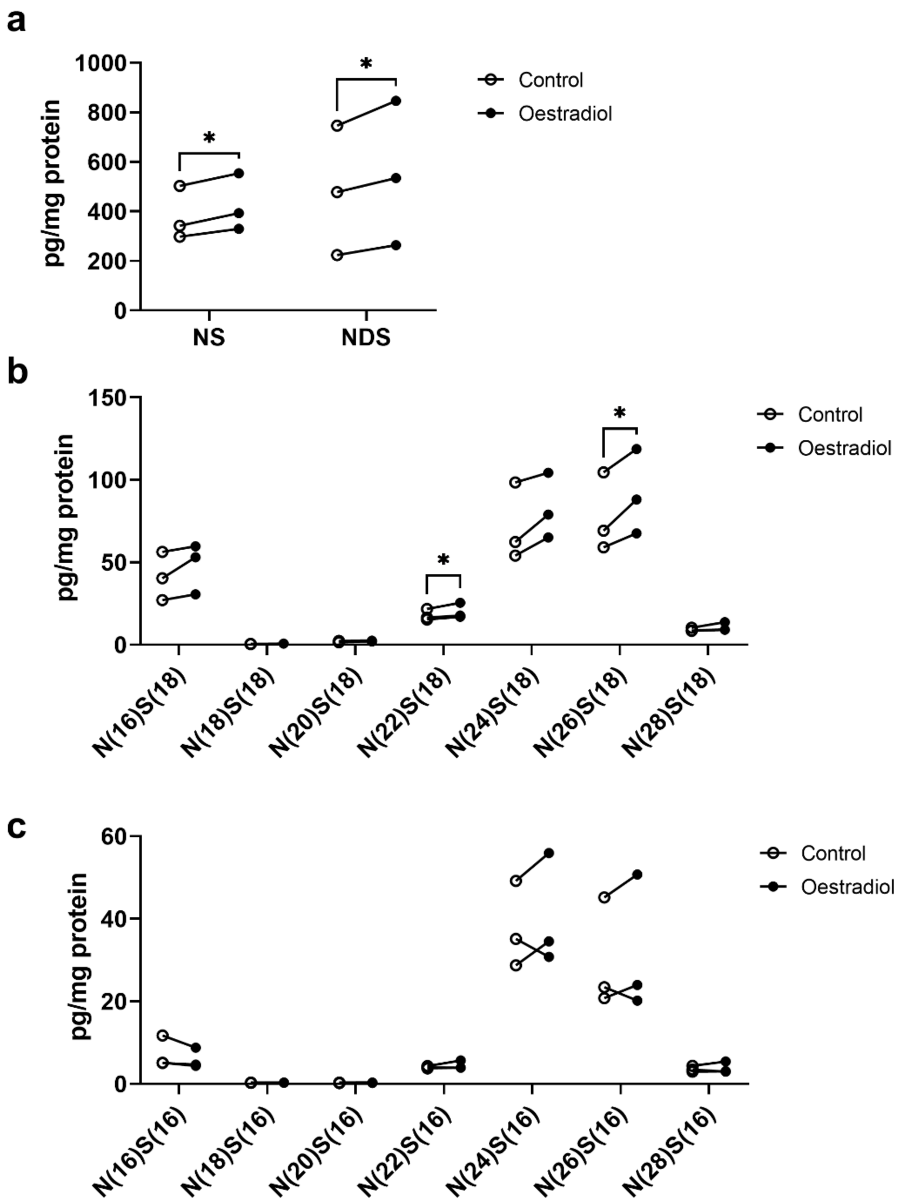

Supplementary Figure S2. Oestradiol provided exogenously to differentiated primary human keratinocytes increases production of CER[NS] and CER[NDS]. Differentiating keratinocytes were treated with oestradiol (10 nM, 72 h). Individual ceramides were then extracted and analysed by ultraperformance liquid chromatography with electrospray ionisation and tandem mass spectrometry and grouped by ceramide class. Total CER[NS] and CER[NDS] are presented (**a**) as well as individual ceramides with a 18-carbon sphingosine base (**b**) and a 16-carbon sphingosine base (**c**). Data are presented as ng/mg protein. *P < 0.05, analysed by ratio paired t-tests, n=3 different keratinocyte donors.

Supplementary Table S1. Solvent gradient for analysis of ceramides and sphingomyelins

Time (min)	Mobile phase A (%)	Mobile phase B (%)	Ramp
0	40	60	6
6	40	60	6
9	4	96	6
20	0	100	6
30	0	100	6
32	40	60	6
40	40	60	6

Supplementary Table S2. Quantitation transitions and parameters for each ceramide analysed

Class	Compound	Precursor ion (*m/z*)	Product ion (*m/z*)	Collision energy (eV)	Cone voltage (V)
ADS	A(24)DS(16)	640.625	256.4	30	30
ADS	A(26)DS(16)	668.656	256.4	30	30
ADS	A(27)DS(16)	682.672	256.4	30	30
ADS	A(24)DS(17)	654.64	270.4	30	30
ADS	A(16)DS(18)-d9	565.603	284.4	30	30
ADS	A(23)DS(18)	654.64	284.4	30	30
ADS	A(24)DS(18)	668.656	284.4	30	30
ADS	A(25)DS(18)	682.672	284.4	30	30
ADS	A(26)DS(18)	696.687	284.4	30	30
ADS	A(22)DS(19)	654.64	298.4	30	30
ADS	A(24)DS(19)	682.672	298.4	30	30
ADS	A(26)DS(19)	710.703	298.4	30	30
ADS	A(18)DS(20)	612.573	312.4	30	30
ADS	A(20)DS(20)	640.625	312.4	30	30
ADS	A(23)DS(20)	682.672	312.4	30	30
ADS	A(24)DS(20)	696.687	312.4	30	30
ADS	A(25)DS(20)	710.703	312.4	30	30
ADS	A(26)DS(20)	724.719	312.4	30	30
ADS	A(24)DS(21)	710.703	326.4	30	30
ADS	A(16)DS(22)	612.573	340.4	30	30
ADS	A(18)DS(22)	640.625	340.4	30	30
ADS	A(22)DS(22)	696.687	340.4	30	30
ADS	A(23)DS(22)	710.703	340.4	30	30
ADS	A(24)DS(22)	724.719	340.4	30	30
ADS	A(18)DS(23)	654.64	354.4	30	30
ADS	A(16)DS(24)	640.625	368.4	30	30
ADS	A(18)DS(24)	668.656	368.4	30	30
ADS	A(16)DS(25)	654.64	382.4	30	30
ADS	A(16)DS(26)	668.656	396.4	30	30
AH	A(24)H(14)	608.562	224.4	30	30
AH	A(25)H(14)	622.577	224.4	30	30

AH	A(26)H(14)	636.593	224.4	30	30
AH	A(28)H(14)	664.624	224.4	30	30
AH	A(24)H(15)	622.577	238.4	30	30
AH	A(26)H(15)	650.609	238.4	30	30
AH	A(28)H(15)	678.64	238.4	30	30
AH	A(22)H(16)	608.562	252.4	30	30
AH	A(23)H(16)	622.577	252.4	30	30
AH	A(24)H(16)	636.593	252.4	30	30
AH	A(25)H(16)	650.609	252.4	30	30
AH	A(26)H(16)	664.624	252.4	30	30
AH	A(27)H(16)	678.64	252.4	30	30
AH	A(28)H(16)	692.655	252.4	30	30
AH	A(22)H(17)	622.577	266.4	30	30
AH	A(24)H(17)	650.609	266.4	30	30
AH	A(26)H(17)	678.64	266.4	30	30
AH	A(28)H(17)	706.671	266.4	30	30
AH	A(16)H(18)-d9	561.587	280.4	30	30
AH	A(20)H(18)	608.562	280.4	30	30
AH	A(22)H(18)	636.593	280.4	30	30
AH	A(24)H(18)	664.624	280.4	30	30
AH	A(25)H(18)	678.64	280.4	30	30
AH	A(26)H(18)	692.655	280.4	30	30
AH	A(27)H(18)	706.671	280.4	30	30
AH	A(28)H(18)	720.687	280.4	30	30
AH	A(29)H(18)	734.702	280.4	30	30
AH	A(30)H(18)	748.718	280.4	30	30
AH	A(24)H(19)	678.64	294.4	30	30
AH	A(26)H(19)	706.671	294.4	30	30
AH	A(28)H(19)	734.702	294.4	30	30
AH	A(24)H(20)	692.655	308.4	30	30
AH	A(25)H(20)	706.671	308.4	30	30
AH	A(26)H(20)	720.687	308.4	30	30
AH	A(27)H(20)	734.702	308.4	30	30
AH	A(28)H(20)	748.718	308.4	30	30
AH	A(26)H(21)	734.702	322.4	30	30
AH	A(24)H(22)	720.687	336.4	30	30
AH	A(26)H(22)	748.718	336.4	30	30
AP	A(22)P(16)	628.588	254.4	30	30
AP	A(24)P(16)	656.62	254.4	30	30
AP	A(26)P(16)	684.651	254.4	30	30
AP	A(24)P(17)	670.635	268.4	30	30
AP	A(26)P(17)	698.666	268.4	30	30
AP	A(16)P(18)-d9	581.629	282.4	30	30
AP	A(22)P(18)	656.62	282.4	30	30
AP	A(24)P(18)	684.651	282.4	30	30
AP	A(25)P(18)	698.666	282.4	30	30

AP	A(26)P(18)	712.682	282.4	30	30
AP	A(22)P(19)	670.635	296.4	30	30
AP	A(24)P(19)	698.666	296.4	30	30
AP	A(26)P(19)	726.698	324.4	30	30
AP	A(20)P(20)	656.62	310.4	30	30
AP	A(22)P(20)	684.651	310.4	30	30
AP	A(24)P(20)	712.682	310.4	30	30
AP	A(25)P(20)	726.698	296.4	30	30
AP	A(26)P(20)	740.713	310.4	30	30
AP	A(24)P(21)	726.698	310.4	30	30
AP	A(24)P(22)	740.713	338.4	30	30
AP	A(26)P(22)	754.729	338.4	30	30
AS	A(24)S(16)	638.609	236.4	30	30
AS	A(25)S(16)	652.625	236.4	30	30
AS	A(26)S(16)	666.64	236.4	30	30
AS	A(27)S(16)	680.656	236.4	30	30
AS	A(24)S(17)	652.625	250.4	30	30
AS	A(26)S(17)	680.656	250.4	30	30
AS	A(16)S(18)-d9	545.561	264.4	30	30
AS	A(23)S(18)	652.625	264.4	30	30
AS	A(24)S(18)	666.64	264.4	30	30
AS	A(25)S(18)	680.656	264.4	30	30
AS	A(26)S(18)	694.672	264.4	30	30
AS	A(27)S(18)	708.687	264.4	30	30
AS	A(24)S(19)	680.656	278.4	30	30
AS	A(26)S(19)	708.687	278.4	30	30
AS	A(28)S(19)	736.718	278.4	30	30
AS	A(23)S(20)	680.656	292.4	30	30
AS	A(24)S(20)	694.672	292.4	30	30
AS	A(26)S(20)	722.703	292.4	30	30
AS	A(22)S(21)	680.656	306.4	30	30
AS	A(24)S(21)	708.687	306.4	30	30
AS	A(23)S(22)	708.687	320.4	30	30
AS	A(24)S(22)	722.703	320.4	30	30
AS	A(25)S(22)	736.718	320.4	30	30
AS	A(26)S(22)	750.734	320.4	30	30
EOH	E(18:2)O(30)H(18)	1010.948	280.4	40	30
EOH	E(18:2)O(31)H(18)	1024.963	280.4	40	30
EOH	E(18:2)O(32)H(18)	1038.979	280.4	40	30
EOH	E(18:2)O(30)H(19)	1024.963	294.4	40	30
EOH	E(18:2)O(32)H(19)	1052.995	294.4	40	30
EOH	E(18:2)O(28)H(20)	1010.948	308.4	40	30
EOH	E(18:2)O(29)H(20)	1024.963	308.4	40	30
EOH	E(18:2)O(30)H(20)	1038.979	308.4	40	30
EOH	E(18:2)O(31)H(20)	1052.995	308.4	40	30
EOH	E(18:2)O(32)H(20)	1067.01	308.4	40	30

EOH	E(18:2)O(30)H(21)	1052.995	322.4	40	30
EOH	E(18:2)O(30)H(22)	1067.01	336.4	40	30
EOP	E(18:1)O(26)P(18)-d9	986.031	282.4	45	30
EOP	E(18:2)O(32)P(18)	1059.006	282.4	45	30
EOP	E(18:2)O(30)P(20)	1059.006	310.4	45	30
EOP	E(18:2)O(31)P(20)	1073.021	310.4	45	30
EOP	E(18:2)O(32)P(20)	1087.037	310.4	45	30
EOP	E(18:2)O(30)P(21)	1073.021	324.4	45	30
EOP	E(18:2)O(28)P(22)	1059.006	338.4	45	30
EOP	E(18:2)O(29)P(22)	1073.021	338.4	45	30
EOP	E(18:2)O(30)P(22)	1087.037	338.4	45	30
EOS	E(18:1)O(26)S(18)-d9	967.973	264.4	45	30
EOS	E(18:2)O(30)S(18)	1012.964	264.4	45	30
EOS	E(18:2)O(30)S(19)	1026.979	278.4	45	30
EOS	E(18:2)O(32)S(19)	1055.011	278.4	45	30
EOS	E(18:2)O(29)S(20)	1026.979	292.4	45	30
EOS	E(18:2)O(30)S(20)	1040.995	292.4	45	30
EOS	E(18:2)O(31)S(20)	1055.011	292.4	45	30
EOS	E(18:2)O(32)S(20)	1069.026	292.4	45	30
EOS	E(18:2)O(30)S(21)	1055.011	306.4	45	30
EOS	E(18:2)O(28)S(22)	1040.995	320.4	45	30
EOS	E(18:2)O(30)S(22)	1069.026	320.4	45	30
NDS	N(24)DS(16)	624.63	256.4	30	30
NDS	N(25)DS(16)	638.646	256.4	30	30
NDS	N(26)DS(16)	652.661	256.4	30	30
NDS	N(24)DS(17)	638.646	270.4	30	30
NDS	N(26)DS(17)	666.677	270.4	30	30
NDS	N(28)DS(17)	694.708	270.4	30	30
NDS	N(16)DS(18)-d9	549.592	284.4	30	30
NDS	N(22)DS(18)	624.63	284.4	30	30
NDS	N(23)DS(18)	638.646	284.4	30	30
NDS	N(24)DS(18)	652.661	284.4	30	30
NDS	N(25)DS(18)	666.677	284.4	30	30
NDS	N(26)DS(18)	680.692	284.4	30	30
NDS	N(27)DS(18)	694.708	284.4	30	30
NDS	N(28)DS(18)	708.724	284.4	30	30
NDS	N(22)DS(19)	638.646	298.4	30	30
NDS	N(24)DS(19)	666.677	298.4	30	30
NDS	N(26)DS(19)	694.708	298.4	30	30
NDS	N(28)DS(19)	722.739	298.4	30	30
NDS	N(20)DS(20)	624.63	312.4	30	30
NDS	N(23)DS(20)	666.677	312.4	30	30
NDS	N(24)DS(20)	680.692	312.4	30	30
NDS	N(25)DS(20)	694.708	312.4	30	30
NDS	N(26)DS(20)	708.724	312.4	30	30
NDS	N(27)DS(20)	722.739	312.4	30	30

NDS	N(28)DS(20)	736.755	312.4	30	30
NDS	N(29)DS(20)	750.771	312.4	35	30
NDS	N(30)DS(20)	764.786	312.4	35	30
NDS	N(24)DS(21)	694.708	326.4	30	30
NDS	N(26)DS(21)	722.739	326.4	30	30
NDS	N(28)DS(21)	750.771	326.4	35	30
NDS	N(30)DS(21)	778.802	326.4	35	30
NDS	N(18)DS(22)	624.63	340.4	30	30
NDS	N(20)DS(22)	652.661	340.4	30	30
NDS	N(22)DS(22)	680.692	340.4	30	30
NDS	N(23)DS(22)	694.708	340.4	30	30
NDS	N(24)DS(22)	708.724	340.4	30	30
NDS	N(25)DS(22)	722.739	340.4	30	30
NDS	N(26)DS(22)	736.755	340.4	30	30
NDS	N(27)DS(22)	750.771	340.4	35	30
NDS	N(28)DS(22)	764.786	340.4	35	30
NDS	N(29)DS(22)	778.802	340.4	35	30
NDS	N(30)DS(22)	792.818	340.4	35	30
NDS	N(22)DS(23)	694.708	354.4	30	30
NDS	N(24)DS(23)	722.739	354.4	30	30
NDS	N(26)DS(23)	750.771	354.4	35	30
NDS	N(28)DS(23)	778.802	354.4	35	30
NDS	N(16)DS(24)	624.63	368.4	30	30
NDS	N(18)DS(24)	652.661	368.4	30	30
NDS	N(20)DS(24)	680.692	368.4	30	30
NDS	N(22)DS(24)	708.724	368.4	30	30
NDS	N(23)DS(24)	722.739	368.4	30	30
NDS	N(24)DS(24)	736.755	368.4	30	30
NDS	N(25)DS(24)	750.771	368.4	35	30
NDS	N(26)DS(24)	764.786	368.4	35	30
NDS	N(27)DS(24)	778.802	368.4	35	30
NDS	N(28)DS(24)	792.818	368.4	35	30
NDS	N(29)DS(24)	806.833	368.4	35	30
NDS	N(30)DS(24)	820.849	368.4	35	30
NDS	N(22)DS(25)	722.739	382.4	30	30
NDS	N(24)DS(25)	750.771	382.4	35	30
NDS	N(26)DS(25)	778.802	382.4	35	30
NDS	N(28)DS(25)	806.833	382.4	35	30
NDS	N(16)DS(26)	652.661	396.4	30	30
NDS	N(18)DS(26)	680.692	396.4	30	30
NDS	N(20)DS(26)	708.724	396.4	30	30
NDS	N(22)DS(26)	736.755	396.4	30	30
NDS	N(23)DS(26)	750.771	396.4	35	30
NDS	N(24)DS(26)	764.786	396.4	35	30
NDS	N(25)DS(26)	778.802	396.4	35	30
NDS	N(26)DS(26)	792.818	396.4	35	30

NDS	N(27)DS(26)	806.833	396.4	35	30
NDS	N(28)DS(26)	820.849	396.4	35	30
NDS	N(22)DS(27)	750.771	410.4	35	30
NDS	N(24)DS(27)	778.802	410.4	35	30
NDS	N(26)DS(27)	806.833	410.4	35	30
NDS	N(26)DS(28)	820.849	424.4	35	30
NH	N(26)H(14)	620.597	252.4	30	30
NH	N(26)H(15)	634.614	238.4	30	30
NH	N(24)H(16)	620.597	224.4	30	30
NH	N(25)H(16)	634.614	252.4	30	30
NH	N(26)H(16)	648.629	252.4	30	30
NH	N(28)H(16)	676.661	252.4	30	30
NH	N(24)H(17)	634.614	266.4	30	30
NH	N(26)H(17)	662.645	266.4	30	30
NH	N(28)H(17)	690.676	266.4	30	30
NH	N(16)H(18)-d9	545.615	280.4	30	30
NH	N(23)H(18)	634.614	280.4	30	30
NH	N(24)H(18)	648.629	280.4	30	30
NH	N(25)H(18)	662.645	280.4	30	30
NH	N(26)H(18)	676.661	280.4	30	30
NH	N(27)H(18)	690.676	280.4	30	30
NH	N(28)H(18)	704.692	280.4	30	30
NH	N(30)H(18)	732.723	280.4	30	30
NH	N(26)H(19)	690.676	294.4	30	30
NH	N(28)H(19)	718.707	294.4	30	30
NH	N(30)H(19)	746.739	294.4	30	30
NH	N(24)H(20)	676.661	308.4	30	30
NH	N(25)H(20)	690.676	308.4	30	30
NH	N(26)H(20)	704.692	308.4	30	30
NH	N(27)H(20)	718.707	308.4	30	30
NH	N(28)H(20)	732.723	308.4	30	30
NH	N(29)H(20)	746.739	308.4	30	30
NH	N(30)H(20)	760.754	308.4	35	30
NH	N(26)H(21)	718.707	322.4	30	30
NH	N(28)H(21)	746.739	322.4	30	30
NH	N(26)H(22)	732.723	336.4	30	30
NH	N(28)H(22)	760.754	336.4	35	30
NP	N(24)P(16)	640.625	254.4	30	30
NP	N(26)P(16)	668.656	254.4	30	30
NP	N(28)P(16)	696.687	254.4	30	30
NP	N(24)P(17)	654.64	268.4	30	30
NP	N(26)P(17)	682.672	268.4	30	30
NP	N(28)P(17)	710.703	268.4	30	30
NP	N(16)P(18)-d9	565.65	282.4	30	30
NP	N(24)P(18)	668.656	282.4	30	30
NP	N(25)P(18)	682.672	282.4	30	30

NP	N(26)P(18)	696.687	282.4	30	30
NP	N(27)P(18)	710.703	282.4	30	30
NP	N(28)P(18)	724.718	282.4	30	30
NP	N(24)P(19)	682.672	296.4	30	30
NP	N(26)P(19)	710.703	296.4	30	30
NP	N(23)P(20)	682.672	310.4	30	30
NP	N(24)P(20)	696.687	310.4	30	30
NP	N(25)P(20)	710.703	310.4	30	30
NP	N(26)P(20)	724.718	310.4	30	30
NP	N(28)P(20)	752.75	310.4	35	30
NP	N(30)P(20)	780.781	310.4	35	30
NP	N(24)P(21)	710.703	324.4	30	30
NP	N(26)P(21)	738.734	324.4	30	30
NP	N(28)P(21)	766.765	324.4	35	30
NP	N(30)P(21)	794.797	324.4	35	30
NP	N(24)P(22)	724.718	338.4	30	30
NP	N(25)P(22)	738.734	338.4	30	30
NP	N(26)P(22)	752.75	338.4	35	30
NP	N(27)P(22)	766.765	338.4	35	30
NP	N(28)P(22)	780.781	338.4	35	30
NP	N(29)P(22)	794.797	338.4	35	30
NP	N(30)P(22)	808.812	338.4	35	30
NP	N(26)P(23)	766.765	352.4	35	30
NP	N(28)P(23)	794.797	352.4	35	30
NP	N(24)P(24)	752.75	366.4	35	30
NP	N(26)P(24)	780.781	366.4	35	30
NP	N(27)P(24)	794.797	366.4	35	30
NP	N(28)P(24)	808.812	366.4	35	30
NP	N(26)P(25)	794.797	380.4	35	30
NP	N(24)P(26)	780.781	394.4	35	30
NP	N(26)P(26)	808.812	394.4	35	30
NS	N(24)S(16)	622.614	236.4	30	30
NS	N(25)S(16)	636.63	236.4	30	30
NS	N(26)S(16)	650.645	236.4	30	30
NS	N(27)S(16)	664.661	236.4	30	30
NS	N(28)S(16)	678.677	236.4	30	30
NS	N(24)S(17)	636.63	250.4	30	30
NS	N(26)S(17)	664.661	250.4	30	30
NS	N(28)S(17)	692.692	250.4	30	30
NS	N(16)S(18)-d9	529.566	264.4	30	30
NS	N(22)S(18)	622.614	264.4	30	30
NS	N(23)S(18)	636.63	264.4	30	30
NS	N(24)S(18)	650.645	264.4	30	30
NS	N(25)S(18)	664.661	264.4	30	30
NS	N(26)S(18)	678.677	264.4	30	30
NS	N(27)S(18)	692.692	264.4	30	30

NS	N(28)S(18)	706.708	264.4	30	30
NS	N(29)S(18)	720.724	264.4	30	30
NS	N(22)S(19)	636.63	278.4	30	30
NS	N(24)S(19)	664.661	278.4	30	30
NS	N(26)S(19)	692.692	278.4	30	30
NS	N(28)S(19)	720.724	278.4	30	30
NS	N(21)S(20)	636.63	292.4	30	30
NS	N(22)S(20)	650.645	292.4	30	30
NS	N(23)S(20)	664.661	292.4	30	30
NS	N(24)S(20)	678.677	292.4	30	30
NS	N(25)S(20)	692.692	292.4	30	30
NS	N(26)S(20)	706.708	292.4	30	30
NS	N(27)S(20)	720.724	292.4	30	30
NS	N(28)S(20)	734.739	292.4	30	30
NS	N(29)S(20)	748.755	292.4	30	30
NS	N(30)S(20)	762.771	292.4	35	30
NS	N(22)S(21)	664.661	306.4	30	30
NS	N(24)S(21)	692.692	306.4	30	30
NS	N(26)S(21)	720.724	306.4	30	30
NS	N(28)S(21)	748.755	306.4	30	30
NS	N(30)S(21)	776.786	306.4	35	30
NS	N(20)S(22)	650.645	320.4	30	30
NS	N(22)S(22)	678.677	320.4	30	30
NS	N(23)S(22)	692.692	320.4	30	30
NS	N(24)S(22)	706.708	320.4	30	30
NS	N(25)S(22)	720.724	320.4	30	30
NS	N(26)S(22)	734.739	320.4	30	30
NS	N(27)S(22)	748.755	320.4	30	30
NS	N(28)S(22)	762.771	320.4	35	30
NS	N(29)S(22)	776.786	320.4	35	30
NS	N(30)S(22)	790.802	320.4	35	30
NS	N(24)S(23)	720.724	334.4	30	30
NS	N(26)S(23)	748.755	334.4	30	30
NS	N(28)S(23)	776.786	334.4	35	30
NS	N(22)S(24)	706.708	348.4	30	30
NS	N(23)S(24)	720.724	348.4	30	30
NS	N(24)S(24)	734.739	348.4	30	30
NS	N(25)S(24)	748.755	348.4	30	30
NS	N(26)S(24)	762.771	348.4	35	30
NS	N(28)S(24)	790.802	348.4	35	30
NS	N(24)S(25)	748.755	362.4	30	30
NS	N(26)S(25)	776.786	362.4	35	30
NS	N(24)S(26)	762.771	376.4	35	30
NS	N(25)S(26)	776.786	376.4	35	30
NS	N(26)S(26)	790.802	376.4	35	30

Supplementary Table S3. Quantitation transitions and parameters for each sphingomyelin analysed

Compound	Precursor ion (*m/z*)	Product ion (*m/z*)	Collision energy (eV)	Cone voltage (V)
SM-d31	734.666	184.1	26	48
SM 32:1	675.536	184.047	20	18
SM 33:1	689.552	184.047	20	18
SM 34:2	701.552	184.047	20	18
SM 34:1	703.568	184.047	20	18
SM 34:0	705.583	184.047	20	18
SM 35:1	717.583	184.047	20	18
SM 36:2	729.583	184.047	20	18
SM 36:1	731.599	184.047	20	18
SM 37:1	745.615	184.047	20	18
SM 38:2	757.615	184.047	20	18
SM 38:1	759.63	184.047	20	18
SM 39:1	773.646	184.047	20	18
SM 40:2	785.656	184.047	20	18
SM 40:1	787.662	184.047	20	18
SM 40:0	789.677	184.047	20	18
SM 41:2	799.662	184.047	20	18
SM 41:1	801.677	184.047	20	18
SM 42:3	811.662	184.047	20	18
SM 42:2	813.677	184.047	20	18
SM 42:1	815.693	184.047	20	18
SM 43:1	829.708	184.047	20	18
SM 44:2	841.708	184.047	20	18
SM 44:1	843.724	184.047	20	18
